# Advances in mapping population and demographic characteristics at small-area levels

**DOI:** 10.1093/ije/dyz179

**Published:** 2020-04-15

**Authors:** Daniela Fecht, Samantha Cockings, Susan Hodgson, Frédéric B Piel, David Martin, Lance A Waller

**Affiliations:** 1 UK Small Area Health Statistics Unit, MRC-PHE Centre for Environment and Health, Imperial College London, St Mary’s Campus, London, UK; 2 School of Geography and Environmental Science, University of Southampton, Southampton, UK; 3 Department of Biostatistics and Bioinformatics, Rollins School of Public Health, Emory University, Atlanta, GA, USA

**Keywords:** Population, census, administrative data, big data, spatio-temporal analysis

## Abstract

Temporally and spatially highly resolved information on population characteristics, including demographic profile (e.g. age and sex), ethnicity and socio-economic status (e.g. income, occupation, education), are essential for observational health studies at the small-area level. Time-relevant population data are critical as denominators for health statistics, analytics and epidemiology, to calculate rates or risks of disease. Demographic and socio-economic characteristics are key determinants of health and important confounders in the relationship between environmental contaminants and health.

In many countries, census data have long been the source of small-area population denominators and confounder information. A strength of the traditional census model has been its careful design and high level of population coverage, allowing high-quality detailed data to be released for small areas periodically, e.g. every 10 years. The timeliness of data, however, becomes a challenge when temporally and spatially highly accurate annual (or even more frequent) data at high spatial resolution are needed, for example, for health surveillance and epidemiological studies. Additionally, the approach to collecting demographic population information is changing in the era of open and big data and may eventually evolve to using combinations of administrative and other data, supplemented by surveys.

We discuss different approaches to address these challenges including (i) the US American Community Survey, a rolling sample of the US population census, (ii) the use of spatial analysis techniques to compile temporally and spatially high-resolution demographic data and (iii) the use of administrative and big data sources as proxies for demographic characteristics.

**Key Messages**

- Temporally and spatially high-resolution population counts are essential for observational health studies at the small-area level to compute rates and risks, and for temporal and spatial health surveillance.
- Approaches to the production of small-area population counts are starting to emerge that go beyond the decennial census still used in most countries.
- Examples include the American Community Survey, the use of spatial analysis techniques to compile temporally and spatially high-resolution demographic data, and the use of administrative and big data sources as proxies for demographic characteristics.
- Such approaches are needed to avoid the increasing inaccuracies in the denominator population seen in traditional census approaches due to residential mobility during inter-censual years.

## Introduction

Geographically referenced population counts are commonly used as the denominator in investigations of diseases in small areas such as those conducted by the UK Small Area Health Statistics Unit (SAHSU). Reliable and time-relevant denominator population counts are vital to calculate rates or risks of disease, and for temporal and spatial health surveillance. Establishing a reliable estimation of detailed, spatially and temporally defined population data is, therefore, crucial for observational or descriptive health studies at small-area scale. This is particularly important for studies of rare non-communicable diseases or covering long periods of time in order to compute stable disease rates. Examples from SAHSU include a study on associations of aircraft noise from Heathrow airport and cardiovascular disease outcomes,^1^ mortality in relation to road traffic noise exposure in London,^2^ respiratory disease patterns in the vicinity of composting sites^3^ and the Environment and Health Atlas for England and Wales.^4^ These studies rely on spatially and temporally detailed denominator population counts, ideally across consistent small-area boundaries. Such information is not readily available from routine sources in most countries.

In 1999, SAHSU published a series of methodological papers on how best to obtain population counts in small areas and discussed the implications of various methods for studies on environment and health.^5^ Approaches, such as the use of ancillary data, e.g. patient registration data, vital statistics and surveys or statistical frameworks, were all found to have their own inherent challenges including under-enumeration, misreporting or misalignment due to geographical boundary changes. Twenty years on, and arguably little has changed. In the UK, as in many other parts of the world, the only reliable individual enumeration of the population remains the decennial census. Some countries have established national registers, most notably Scandinavian countries where individuals are being tracked across the life course using a personal identity number.^6^ These are, however, exceptions and the census is still the most widely used source of population information.

Nonetheless, there have been significant advances in census-taking internationally during the last 20 years and, since 2010, alternative approaches to the production of small-area population counts have started to gather pace in many countries.

At a time when demands of spatial analyses are increasingly sophisticated and greater spatial and temporal precision is required, conventional data collection mechanisms are becoming increasingly difficult to implement. This paper provides an overview of the challenges faced by contemporary mapping and spatial analysis of population data, from a public health perspective. Drawing on examples from the UK and USA, we highlight some of the approaches that are currently being implemented focusing on sophisticated spatial statistics and big data solutions to address the shortcomings and uncertainties of conventional population enumeration. We focus on three major methodological strands of obtaining time-relevant small-area population estimates: (i) the American Community Survey (ACS), a rolling sample of the US population census, (ii) the use of spatial analysis techniques to compile temporally and spatially high-resolution demographic data and (iii) the use of administrative and big data sources as proxies for demographic characteristics.

## Censuses and small-area geographies

Whereas the enumeration of the population can be traced back more than two centuries (the first census took place in 1790 in the USA and 1801 in the UK), the digital processing of census data and spatial referencing were not generally undertaken until the 1970 US Census, when the Dual Independent Map Encoding (DIME) system was introduced.^7^ This structured geographical small-area reference system was subsequently developed as the Topologically Integrated Geographic Encoding and Referencing (TIGER) system. Successive census rounds internationally have seen steady enhancements in the sophistication of geographical referencing, together with increasing integration with national spatial data infrastructures. Traditional census enumeration has evolved from being undertaken with support from paper mapping to geographic information system (GIS) management of the residential address.^8^ In the UK, digital boundaries for the smallest spatial units were produced retrospectively for the 1981 and 1991 census (enumeration districts) and as part of the census process itself from 2001 (census output areas).

Well-conducted contemporary censuses provide an enormously rich data resource, comprising not only carefully designed, high-quality demographic and socio-economic data, but also a supporting framework of small-area boundaries as well as address, postcode or ZIP code listings. In addition, they provide mapping tools, specialist datasets including commuting and migration data and microdata samples. Although proceeding at different speeds, many developed countries have now integrated census data infrastructures with full listings of addresses. These address-based infrastructures assist with the delivery of census questionnaires (whether by post or by hand) and provide the highest resolution means of georeferencing both census returns and any other address-referenced data to the census geographical units;^9^ examples include routinely collected health data such as births and deaths from the Office for National Statistics in the UK, or high-resolution modelled exposure data.^10^

The intervening decades have seen continuous improvement in many aspects of census processing but also brought new challenges. Within the context of traditional census design, particular challenges are (i) increasing expectations of users, particularly for greater timeliness of data, (ii) declining willingness of population members to participate, perhaps associated with declining levels of trust in government, (iii) reflecting the complexities of contemporary society, such as complex family living arrangements and (iv) the substantial costs faced by national statistical organisations in an era of widespread cuts in public expenditure.

Since 2000, and with greater impetus since 2010, censuses have started to change dramatically as different countries have adopted a variety of alternative strategies to overcome these challenges.^11^^,^^12^ Responses include the replacement of full censuses with a mixed model of rolling surveys and short-form census (as in the USA), traditional census questionnaires that are increasingly based on internet data collection (such as in the UK, Australia, Canada and New Zealand)^13^ and the replacement of a statistical system firmly based on the census with an increasing diversity of administrative and new forms of data (notably in countries such as Austria where some form of population register is present).^14^ This replacement, or reduced prominence of, censuses should not be read as a necessarily negative narrative, as many new data sources offer the potential to provide increased timeliness and perhaps even to move beyond the census model in terms of finding data frameworks able to better represent modern societies. Just as many health data may be seen as direct products of administrative systems, so new denominator data sources are now being constructed by the amalgamation of administrative data series.

## American community survey

In 2005, the US Census Bureau introduced the ACS as an ongoing, monthly, rolling survey of approximately 250 000 US households, conducted alongside decennial censuses. The ACS uses the monthly data to provide annual population estimates at the national, state and county levels. Population estimates at the census tract level (a subdivision of county) are provided as 5-year rolling averages (i.e. annual estimates are based on 5 years of data) for the years 2009–16. The ACS is carefully designed and includes some oversampling of areas with low population size and/or history of low response rates. It includes data on variables such as education, occupation and income. These were historically collected on the so-called ‘Long Form’ of the US Census, a 1-in-6 household survey that was discontinued following the 2000 decennial US Census. ACS-based population estimates were first released in 2008 based on data collected in the years 2005–07. Importantly, the ACS reports population estimates and associated margins of error for each reporting unit (e.g. state, county or census tract).

The ACS data provide a new source for population denominator data in small-area health studies, and recent studies are beginning to explore the spatial structure of the estimates and of their reported uncertainty (as summarized in the margin of error terms). Spielman *et al.*^15^ note that the ACS represents ‘a nuanced and sensitive set of compromises’ defining small-area population data for over 200 000 block groups and 70 000 census tracts across the US. These smaller geographic areas tend to have smaller sample sizes, hence greater uncertainty. The margins of error for census tract data are, on average, 75% higher than tract data from for the previous 2000 census.^15^ Challenges of statistical precision increase when further subdividing census counts into demographic subgroups, as illustrated by Spielman and Folch^16^ who report 72% of census tracts with margins of error higher than the estimated number of children <5 years old living in poverty. These uncertainties are characterized by statistical and geographic patterns in the reported uncertainty (for example, low-income tracts tend to have higher uncertainty in their population estimates) and high spatial correlation in these levels of uncertainty.^15^

Small-area epidemiologic analyses in the USA are beginning to use ACS population estimates to define populations at risk. Many analyses, however, ignore the uncertainty associated with these estimates, potentially leading to non-differential mis-classification and bias in estimations of association and prediction of local trends. The nature and direction of such biases depend or whether one seeks unbiased estimates of the aggregate level (ecological) effect or whether one seeks unbiased estimates of the individual level effect. In the former, symmetric measurement error may diminish significance due to increased variation in estimates. In the latter, the combination of data aggregation and measurement error can bias estimates away from the null.^17–19^ To address this issue, Finucane *et al.*^20^ and Mercer *et al.*^21^ proposed incorporating the reported design-based uncertainty within Bayesian hierarchical disease mapping models for small-area health studies. Briefly, suppose we define *Y_i_* as the number of cases of disease observed in region *i* and *n_i_* as the (true, unknown) population at risk. The typical disease mapping model proposes:

$$Y_{i}|\beta ,\theta ,\varphi \;∼\;\mathrm{Poisson}\left(E_{i}\exp\left(X^{'}\beta +\theta_{i\;}+\;\phi_{i}\right)\right),$$

where

$$\begin{array}{l} E_{i}=\;\lambda n_{i},\;\\ \theta_{i}∼N\left(0,\;\sigma^{2}\right),\;i=1,\ldots ,I,\\ \varphi ∼N\left(0,\;\Sigma \right) \end{array}$$

$E_{i}$ denotes the expected number of cases in region i, λ denotes the reference population, θ denotes spatially unstructured random effects, φ denotes spatially structured random effects, and Σ denotes a spatial correlation matrix. Regrouping the first line, we find

$$Y_{i}|\beta ,\theta ,\varphi ∼\mathrm{Poisson}(\exp\left({\log\left(\lambda \right)+X}^{'}\beta +(\log\left(n_{i}\right)+\theta_{i}+\varphi_{i})\right)),$$

where *n_i_* is no longer assumed to be fixed, but rather an estimate with a reported margin of error (hence, a derivable variance). Potential estimates of *n_i_* include those from the 2010 census or from the ACS. Assuming sufficient sample sizes, we could model the ACS estimates as unbiased normal random variables with variance defined by the design-based margin of error, i.e. replace *n_i_*with

$${\hat{n}}_{\mathrm{ACS},i}\;∼\;N\left(n_{i},\;\sigma_{\mathrm{ACS},\;i}^{2}\right).$$

Furthermore, Spielman *et al.*’s observation of spatial correlation among ACS estimates suggests extension to

$${\hat{n}}_{\mathrm{ACS}}\;∼\;N\left(n,\;\Sigma_{\mathrm{ACS}}\right),$$

where $\Sigma_{\mathrm{ACS}}\;$denotes a spatial covariance matrix.^14^

Such first steps are not quite ready for direct implementation as we estimate population distribution within the margins of errors via ${\hat{n}}_{\mathrm{ACS},i}$ and do not observe the population at risk $n_{i}.$ Promising results appear in Finucane *et al.*^20^ and Mercer *et al.*^21^ and future studies are needed to further explore the full impact of estimation uncertainty on population estimates and consequent disease studies.

**Figure 1.**
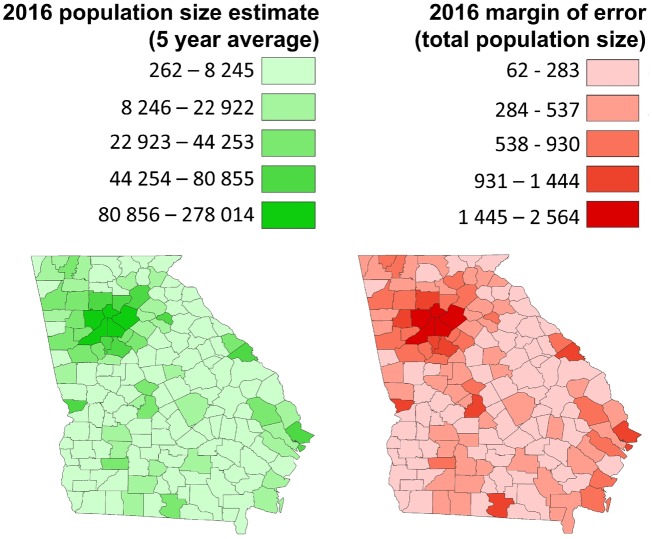
Estimated 2016 population counts in quintiles (left) and associated margins of error (right) from the US American community survey for counties in the state of Georgia, USA.

As an example, Figure 1 illustrates the estimated 2016 population counts and associated margins of error from the ACS for counties in the state of Georgia within the USA. Both choropleth maps are shaded by quintiles of values and, not surprisingly, areas with the highest counts also have the highest margins of errors. We note that the estimates and the estimation error are spatially autocorrelated and that high values (and errors) are concentrated in the urban areas within Georgia.

## Spatio-temporal population modelling: procedures for estimating population at small areas

### The SAHSU population approach

The work of SAHSU strongly depends on spatially and temporally detailed population estimates, especially for studies spanning many decades and therefore many census years.^22^ This brings with it the inherent complexity of changing census geographies used for reporting and dissemination of UK census data from different censuses, which typically reflect changing land use, housing developments and socio-economic characteristics in the local area.

As in most countries, the UK censuses provide population data at a high spatial resolution but with a low temporal resolution (every 10 years). An alternative source of information is the annual population estimates produced by the Office for National Statistics, National Records for Scotland and the Northern Ireland Statistics and Research Agency that have been available since 1981 at local authority level (average population ∼140 000). This is at a much lower geographical resolution than used by the census. These mid-year resident population estimates are disseminated by sex and 5-year age groups. Mid-year population in the inter-censual years are estimated by rolling forward the census base taking account of natural changes such as births and deaths as well as migration. These estimates are revised after each census to account for cohort changes between the census years to avoid step changes in the population estimates series.^23^ Since the 2001 census, these mid-year population estimates are available at the census output area level. Comparable data are lacking for the pre-2001 period, which causes problems for investigations of historic or long-term exposures.^24^

To address these shortcomings, SAHSU developed a spatio-temporal model to estimate historic population data for Great Britain by 5-year age groups and sex from 1981 to 2001 at a consistent geographical level, the 2011 census output areas. This process made use of publicly available population and geographical data (Table 1) and harmonized those across census boundary changes. To do so, we established a reproducible and automated set of procedures in *PostGIS*, a spatial database extender for PostgreSQL.

**Table 1.** Routinely available population data sources in the UK over the last three decades used by SAHSU for the estimation of population at small areas

	National census	Mid-year estimates
Source	UK Data Service Census Support: http://infuse.ukdataservice.ac.uk/	England and Wales: Office for National Statistics
		https://www.ons.gov.uk/peoplepopulationandcommunity/populationandmigration/populationestimates
	http://casweb.ukdataservice.ac.uk/	
Spatial resolution	Enumeration districts for 1981 and 1991 census; average population ∼400	Local authority district; average population ∼140 000
	Census output areas for 2001 and 2011 census; average population ∼250	
Temporal resolution	Decennial: 1981, 1991, 2001, 2011	Annual: 1981–2017

We combined data from the censuses and Office for National Statistics mid-year estimates to calculate 5-year age group and sex-specific annual population for each census output area. In a first step, we harmonized the different census geographies to 2011 census geography using postcode weighting as described in detail by Briggs *et al.*^24^^,^^25^ We modelled the census population *C* to 2011 census output area (COA11) *j* for census years *x* (1981, 1991, 2001, 2011) and annual mid-year population estimates *MYE* to 2011 Local Authority District (LAD11) *n* for years *i* (1981–2017). For the four census years, we then calculated a COA11/LAD11 ratio $R_{\mathrm{kjx}}$

$$R_{\mathrm{kjx}}=\frac{C_{\mathrm{kjx}}}{{\mathrm{MYE}}_{\mathrm{knx}}},$$

where $C_{\mathrm{kjx}}$ is the census population for age/sex group *k*, census year *x* and COA11 *j*, and ${\mathrm{MYE}}_{\mathrm{knx}}$ the mid-year population estimate for age/sex group *k*, LAD11 *n* and census year *x.*

Between census years *x* and *x−10*, we then model a ratio change ${\mathrm{RC}}_{\mathrm{kji}}$ as follows:

$$\begin{array}{c} {\mathrm{RC}}_{\mathrm{kji}}=\mathrm{0.1}*\left(R_{\mathrm{kxj}}-R_{\mathrm{kx}-10j}\right),\\ i=1,\;\ldots .,\;10 \end{array}$$

Finally, to compute the COA11 level annual population estimates *MYE_kji_*, we use linear interpolation for intercensal years by applying *RC_kij_* as follows:

$$\begin{array}{c} {\mathrm{MYE}}_{\mathrm{kji}}=\;{\mathrm{MYE}}_{\mathrm{kni}}*\left(R_{\mathrm{kjx}}+{\mathrm{RC}}_{\mathrm{kji}}\right)\\ {\mathrm{MYE}}_{\mathrm{kji}+1}=\;{\mathrm{MYE}}_{\mathrm{kni}+1}*\left(R_{\mathrm{kjx}}+{\mathrm{RC}}_{\mathrm{kji}+1}\right)\\ \begin{array}{c} \ldots \\ {\mathrm{MYE}}_{\mathrm{kji}+10}=\;{\mathrm{MYE}}_{\mathrm{kni}+10}*\left(R_{\mathrm{kjx}+10}+{\mathrm{RC}}_{\mathrm{kji}+10}\right) \end{array} \end{array}$$

**Figure 2.**
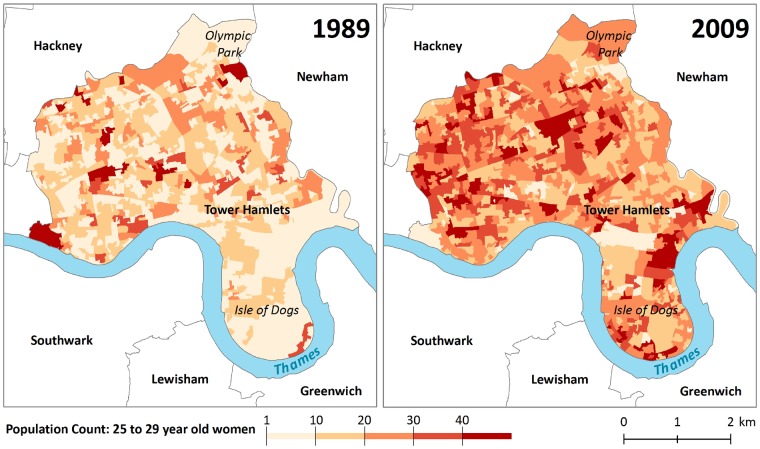
Population estimates (women 25–29 years old) for Census Output Areas 2011 in the London Borough of Tower Hamlets in 1989 (SAHSU estimates) and 2009 (Office for National Statistics mid-year estimates). Tower Hamlets is in the East of London and has seen considerable urban redevelopment over the last decades, such as the redevelopment of the Isle of Dogs from an abandoned industrial site to a leading financial centre, as well as the redevelopment around the 2012 Olympic side in Stratford. Contains National Statistics data © Crown copyright and database right 2012, 2017.

SAHSU’s 5-year age group and sex-specific population estimates span almost 40 years (1981–2017), a period that has seen great changes in the demographic composition across the UK. Our method improves on previous annual population estimates in that estimates are at a high spatial resolution that is consistent across all years. Such data is important for spatial studies spanning many years/decades as our data allows easy comparison of demographic patterns over time and space (see Figure 2). Modelled population estimates are available upon request from the authors.

### The Population247 approach

The advances in census-taking and the use of alternative data sources potentially provide opportunities for obtaining higher resolution spatio-temporal population estimates, whereas methods for consolidating population estimates onto consistent geographies through time provide stable units for analysis. In many instances, however, the high levels of spatio-temporal resolution ideally required by environmental and health studies are still a long way from being achieved. A particular challenge is establishing how population varies over short timescales, such as time of day, day of week or season. Data sources that provide signals of population activity across different temporal cycles, such as diurnal, weekly or seasonal, do exist or are becoming available, but the real difficulty lies in being able to integrate and calibrate these diverse data sources of varying spatial and temporal precision and accuracy in order to provide reliable population denominator data. The University of Southampton ‘Population247’ approach (http://pop247nrt.geodata.soton.ac.uk/) provides a framework, methodology and software (SurfaceBuilder247) for integrating various data sources, such as census, administrative and big data, in order to provide high-resolution spatio-temporal population estimates.^26^ The Population247 approach involves compilation of an extensive data library containing information on locations, demographics, catchment areas, spatial extents and time profiles of the places where people may be located at different times of day, week, season etc. These data are then integrated to produce time-specific estimates of population at a high spatial resolution (e.g. 100–200 m grid squares) for any time of day, date and year for which data are available.

**Figure 3.**
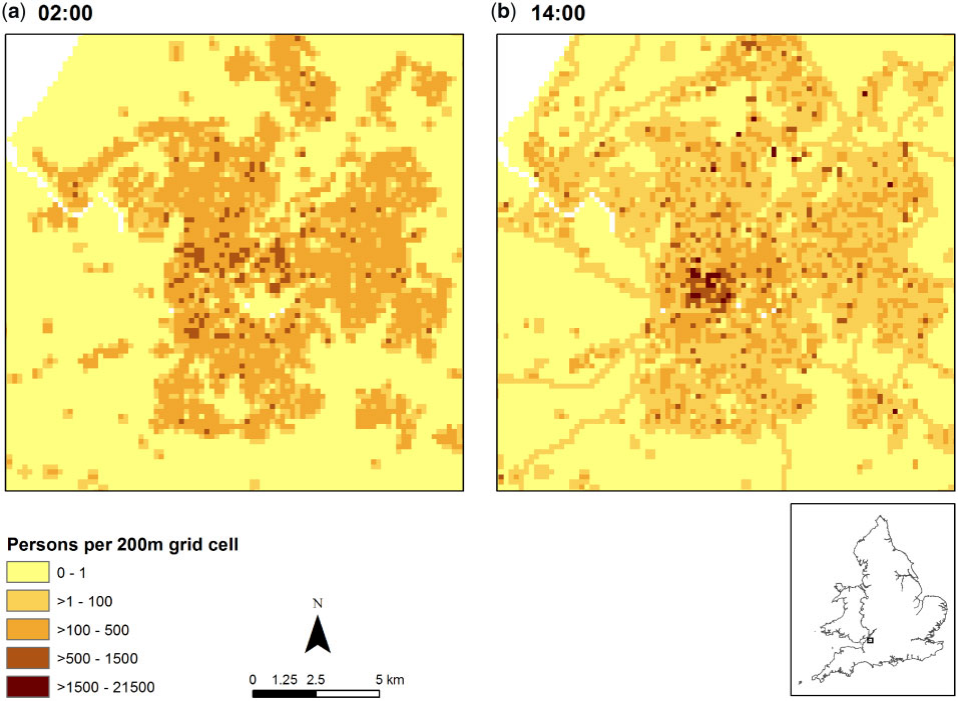
Total population in 200 m grid cells at different time frames: (a) 02:00 and (b) 14:00 hours in Bristol, UK, on a typical weekday during school and university term-time in 2011.

Figure 3 presents illustrative outputs from SurfaceBuilder247, showing total population present in 200 m grid cells at the time frames (a) 02:00 and (b) 14:00 hours for Bristol, UK (∼500 000 people), on a typical weekday during school and university term-time in 2011. At 02:00 hours, population is largely concentrated in residential areas, with few people in the city centre or in transit on the roads: essentially, this is similar to the 2011 residential distribution depicted by the 2011 census at small-area level, but further refined by the use of a 200 m grid. By contrast, at 14:00 hours, population is much reduced in these residential areas and there are much greater concentrations of population within the city centre, in places of work and education and on the road network. These 02:00 and 14:00 hours models are just two of many that could be produced: the key benefit of the Population247 approach is that it is not constrained to specific pre-determined time slices or categorizations of time, but is instead inherently flexible in this respect.

Population247 is therefore a key advancement in the move away from a traditional residential, night-time, decennial-based static census model to more spacetime specific dynamic population estimates. As such, it gets much closer to the spatio-temporal granularity required by small-area environment and health studies, for example, to assign hourly exposures based on daily aggregated activity patterns, capturing home, work and in-transit environments. The framework is extensible, so that as more detailed information becomes available, these can be integrated into the model, with subsequent improvements in the precision and accuracy of estimates at those spaces/times. Current Population247 research is focused on developing methods for harvesting, calibrating and integrating new and emerging forms of data (such as footfall or traffic sensors and data served up via Application Program Interfaces), with existing forms of data (such as census and administrative data), in order to produce enhanced spacetime specific population estimates for improved decision-making and policy formulation in the health, emergency response and national security sectors.

## Alternative data sources as proxies for demographic characteristics

### Administrative data

**Figure 4.**
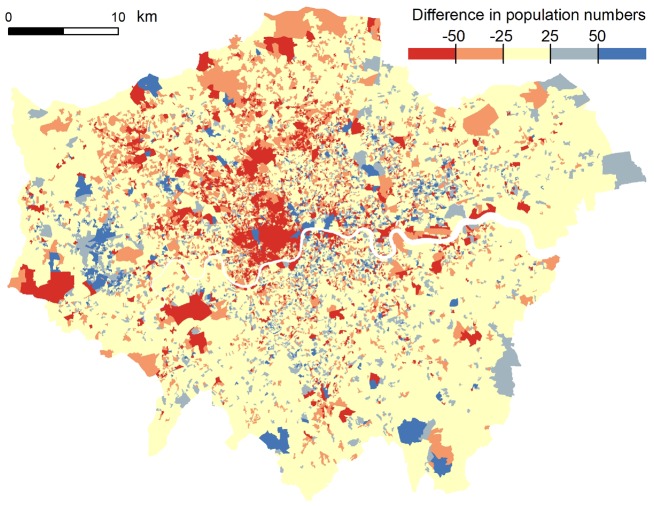
Difference between admin-based (Statistical Population Database v2.0) and census-based total population estimates for Census Output Areas, Greater London, 2011. Red hues highlight areas where the admin-based database over-estimates census-based population, blue hues highlight areas where the admin-based database under-estimates census-based population. Contains National Statistics data © Crown copyright and database right 2012, 2017.

A third means of addressing the challenges faced by censuses is to seek entirely different sources for the traditional census variables, or indeed find new approaches. The first potential source domain, and the one that has been most widely pursued to date, is the enormous range of administrative information that is collected by governments for the operation of systems such as health care, property registration, and taxation and benefits. These sources provide extensive coverage of the population, are generally referenced at the address level and have the potential to be integrated to provide a complete multivariate population database. This approach is much more tractable in countries with a population registration system such as Finland and The Netherlands, due to the presence of systematic individual citizen identifiers to facilitate direct matching of the source datasets. This has led the transition from traditional census-taking in these countries.^27^ The UK’s three national statistical organisations (Office for National Statistics, National Records of Scotland and Northern Ireland Statistics and Research Agency) hold separate but coordinated censuses and all are moving towards a 2021 design that is a combination of conventional census enumeration with a significant emphasis on internet data collection, accompanied by parallel construction of an administrative data census model.^28^ These two models are being developed in parallel, allowing the administrative data to potentially replace or enhance the more conventional census in 2021, while also being developed with the intention that it should form a replacement data collection model post-2021. This work is already beginning to yield experimental data series produced from administrative sources, with successive extensions and enhancements scheduled on an annual basis.^29^ Such data sources appear to offer the potential both to replace some census questions, such as the number of households or the number of rooms in each dwelling (which can be obtained from administrative data about property registration), and to include new topics such as income, which have not previously been covered by the traditional census questionnaire in the UK.^30^^,^^31^ The continuous nature of the administrative data sources makes possible the extraction of regular small-area population count updates, with far greater frequency than the conduct of traditional decennial census enumeration. Figure 4 illustrates differences between 2011 census estimates of total population and a current version of experimental statistics^29^ based on administrative sources combining the National Health Service register, school census, higher education, taxation and national insurance systems. The map units are census output areas (mean population 326) for London. In most areas, the administrative data provide a close approximation to the census counts, but there are some large and spatially concentrated differences. A preliminary investigation suggested that these related primarily to students, armed forces personnel or residents of communal establishments who are recorded differently for census and administrative purposes, but further analyses are needed to better understand the origin of such differences.

### Big data

The second source domain is the ever-increasing range of big data sources, usually not counts of people, but automated records of events, such as the location of mobile devices or social media activity, that can potentially be interpreted as proxies for the presence of human activity and behaviours.^32^ Initial examples have particularly focused on population dynamics, e.g. the use of mobile phone location data to estimate commuting flows^33^ and migration movements,^34^ the first of which are not readily identifiable from administrative data sources. The locational precision varies according to the technologies employed, with locations estimated from the mobile phone network having much lower accuracy than those directly recorded by Global Positioning System trackers in mobile devices. A key challenge with these data types is their calibration with the population concept that is desired to be measured. Deville *et al.*^35^ found quality levels that offer a promising means of estimating population counts from mobile phone data in low-income countries where more formal sources are not available. Such data is also commonly held by private companies such as network providers and challenges arise in obtaining such data for research purposes. In addition, social media data is starting to emerge as a means of estimating demographic characteristics and migration patterns.^36^ Issues of incomplete and biased population coverage, however, mean that such methods are currently still a long way from the quality levels stipulated by national statistics institutions to permit full replacement of the census in countries such as the UK.

## Conclusion

As these examples show, advanced spatio-temporal high-resolution approaches to mapping and modelling population and demographic information are starting to emerge, combining new types of data and analytical innovations. Although these are mostly still at development stages and methods to apply them in health studies need further refinement (see the example above of the ACS in the USA), such advances will prove crucial for future disease mapping and spatial epidemiological studies, particularly those conducted at small-area level.

Such data are urgently needed for epidemiological studies to avoid the increasing inaccuracies in the population denominators derived from traditional census approaches due to residential mobility in the underlying population during inter-censual years. Research has been undertaken to understand the extent and impact of residential mobility in specific subgroups of the population. Studies suggest that we can anticipate considerable migration in some sub-groups of the population (e.g. 7–32% of pregnant women move during pregnancy).^37^^,^^38^ Although many of these moves are local and happen within a city, this is still likely to introduce error into the denominator population in studies undertaken at the small-area level. Studies in the USA, reviewed by Bell and Belanger^37^ have shown that more than 50% of women who moved during pregnancy were likely to remain in the same county, but only 6.2% stayed within the same census tract. Furthermore, there is likely to be differential migration by age and deprivation.^39^ Analyses reliant on data that no longer reflect the actual denominator population will be biased, compromising our ability to correctly identify risk factors for disease and/or control for important confounding variables.^40^

The use of administrative and auxiliary data to supplement or replace the traditional census might overcome some of the issues associated with the increasingly error-prone denominator data with increasing time since the census. However, marginalized groups, including frequent movers and those living in temporary accommodation or communal establishments, are not well captured in these data. The data also fails to account for refugees and sudden population movements triggered by conflicts or environmental disasters. Administrative data may also contain duplicate entries that can be challenging to resolve. For example, according to the UK National Health Service National Strategic Tracing Service records,^39^ 3% of women who moved during pregnancy were registered with general practitioners (GP) under different residential addresses for more than 1 month, the average time of overlap being 14 months, with some women remaining registered at a GP using their previous address for more than 4 years.

The validation of these alternative data sources therefore remains an issue and new approaches are needed to account for uncertainties and under- or over-enumeration in population estimates. As illustrated by SAHSU’s work to standardize geographies and data from multiple censuses for long-term epidemiological studies, similar approaches will need to be developed to account for the inclusion of new alternative data sources. In relation to uncertainty, methods, such as those introduced for the US ACS taking account of margins of errors in disease mapping, provide a good starting point. It will be, however, important to understand the extent to which the same underrepresented populations continue to be underrepresented in new data sources, e.g. through disengagement from administrative processes or digital exclusion, and to make appropriate analytical adjustments.

Studies are starting to emerge that move beyond static population estimates by producing time-varying population estimates at a higher than annual temporal resolution. Such approaches, e.g. the Population247 project described above, combine new, emerging and existing datasets to produce enhanced time-specific population data. These temporally more refined population estimates can be used to inform decision-making and policy formulation in relation to emergency responses and have already been applied in assessing population exposure to natural hazards.^41^^,^^42^ They could also be used to produce enhanced exposure estimates, e.g. if overlaid with air pollution maps of hourly-, daily- or weekly-averaged concentrations. This would pick up short-term peaks of exposures that are lost if aggregated over larger time intervals and could potentially provide a near individual-level exposure for whole populations needed to study health effects of low exposures.

Such advances in estimating spatially and temporally population estimates will open new avenues for small-area studies of health and disease in the future. A first step, however, will need to be the estimation of uncertainty for all these methods in order to propagate uncertainty in population estimates through to uncertainty in epidemiologic risk estimates.

## Funding

This work was supported by the UK Small Area Health Statistics Unit which is funded by Public Health England as part of the Medical Research Council/Public Health England Centre for Environment and Health, funded also by the UK Medical Research Council (MR/L01341X/1), Economic and Social Research Council Awards RES-062–23-0081, ES/P010768/1 which support the University of Southampton Population247 research, and in part by grant R01HD092580 from the US Eunice Kennedy Shriver National Institute of Child Health & Human Development (NICHD). The views represent those of the authors and do not necessarily represent those of Public Health England or the US NICHD.
